# Integration of proteomic and metabolomic characterization in atrial fibrillation-induced heart failure

**DOI:** 10.1186/s12864-022-09044-z

**Published:** 2022-12-01

**Authors:** Haiyu Zhang, Lu Wang, Dechun Yin, Qi Zhou, Lin Lv, Zengxiang Dong, Yuanqi Shi

**Affiliations:** 1 Key Laboratory of Cardiovascular Disease Acousto-Optic Electromagnetic Diagnosis and Treatment in Heilongjiang Province, the First Affiliated Hospital, Harbin Medical University, 23 Youzheng Street, Nangang District, Harbin, 150001 China; 2 Department of Cardiology, the First Affiliated Hospital, Harbin Medical University, 23 Youzheng Street, Nangang District, Harbin, 150001 China; 3 Research Management Office, the First Affiliated Hospital, Harbin Medical University, 23 Youzheng Street, Nangang District, Harbin, 150001 China

**Keywords:** Atrial fibrillation, Heart failure, Proteomics, Metabolomics, Biomarkers

## Abstract

**Background:**

The exact mechanism of atrial fibrillation (AF)-induced heart failure (HF) remains unclear. Proteomics and metabolomics were integrated to in this study, as to describe AF patients’ dysregulated proteins and metabolites, comparing patients without HF to patients with HF.

**Methods:**

Plasma samples of 20 AF patients without HF and another 20 with HF were analyzed by multi-omics platforms. Proteomics was performed with data independent acquisition-based liquid chromatography-tandem mass spectrometry (LC-MS/MS), as metabolomics was performed with LC-MS/MS platform. Proteomic and metabolomic results were analyzed separately and integrated using univariate statistical methods, multivariate statistical methods or machine learning model.

**Results:**

We found 35 up-regulated and 15 down-regulated differentially expressed proteins (DEPs) in AF patients with HF compared to AF patients without HF. Moreover, 121 up-regulated and 14 down-regulated differentially expressed metabolites (DEMs) were discovered in HF patients compared to AF patients without HF. An integrated analysis of proteomics and metabolomics revealed several significantly enriched pathways, including Glycolysis or Gluconeogenesis, Tyrosine metabolism and Pentose phosphate pathway. A total of 10 DEPs and DEMs selected as potential biomarkers provided excellent predictive performance, with an AUC of 0.94. In addition, subgroup analysis of HF classification was performed based on metabolomics, which yielded 9 DEMs that can distinguish between AF and HF for HF classification.

**Conclusions:**

This study provides novel insights to understanding the mechanisms of AF-induced HF progression and identifying novel biomarkers for prognosis of AF with HF by using metabolomics and proteomics analyses.

**Supplementary Information:**

The online version contains supplementary material available at 10.1186/s12864-022-09044-z.

## Introduction

As the most common arrhythmia in clinical practice, atrial fibrillation (AF) happens with complex pathophysiology, that causes adverse consequences of death, stroke and heart failure (HF) [1]. Although the incidence rate of stroke in AF has been decreasing due to the use of oral anticoagulation [2], there has been no obvious change in the incidence or prevalence rate of HF over the past decades [3]. The cumulative incidence rate of HF in AF was reported as 20% at 5 years, which was higher than the risk of stroke [4]. HF is considered to be the most general adverse event caused by AF following hospitalization and death [5]. When AF and HF coexist in patients, the prognosis is poorer than either single condition [6, 7]. It was confirmed that AF with HF shows the risk of death 3.4 fold higher than those patients of AF without HF [4]. Common risk factors and pathophysiological processes have been observed in AF and HF [8]. Conventional risk factors such as advancing age, diabetes, history of cardiovascular disease and hypertension have been shown to predict AF patients for the development of HF [9]. AF has been proposed to drive the development of incident left ventricular dysfunction and cause HF [10]. Despite there were some understandings of the pathophysiological processes in AF complicated with HF, the definite mechanisms of HF in AF were still unclear [10]. Thus, there is an unmet clinical need to investigate molecular mechanisms in AF with HF and probe into reliable biomarkers and drug therapeutic targets for effective treatments.

Proteomics and metabolomics are “omics” techniques that explore the whole proteome and metabolome delivered in specific biological samples [11, 12]. Not as genetics, proteomics and metabolomics concern more on the phenotype of diseases and its progression [13]. The liquid chromatography-tandem mass spectrometry (LC-MS/MS) technology has high sensitivity and can analyze the expression patterns of hundreds to thousands of proteins and metabolites in samples [14, 15], so as to provide useful information for biomarkers detection and metabolic pathways [16]. Integrative analysis that combined proteome and metabolome profiling has been shown to provide novel insights in the understanding of the mechanism of disease occurrence and development [17]. For better study of AF, proteomics and metabolomics techniques have been combined to facilitate that. Hu et.al found that patients relative to sinus thythm with AF show dysregulated proteins and metabolites with the technology of proteomics and metabolomics [18]. Li et.al identified the molecular mechanisms and possible biomarkers for chronic AF in mitral valve disease by iso baric tags for relative and absolute quantitation (iTRAQ)-based proteomics and gas chromatography-mass spectrometer (GC-MS)-based metabolomics [19]. To date, few studies have investigated potential biomarkers and related molecular mechanisms in AF patients with and without HF by combined proteomics and metabolomics approach.

Using metabolomics and proteomics of LC-MS/MS platform, we described the dysregulation of metabolites and proteins in AF patients with and without HF. This study also provided candidate biomarkers for identification of AF with HF, providing available treatment strategies for secondary prevention of HF in AF.

## Methods and materials

### Patient recruitment and sample collection

With the approvement by the Ethics Committee of the First Affiliated Hospital of Harbin Medical University (Harbin, China, IRB-AF202255), 20 persistent AF patients with / without HF respectively were chosen into the study during 2015. 02 and 2017. 01. Patients diagnosed of persistent AF and HF were firstly excluded out of the study by cardiologist under such circumstances: malignant tumor, simultaneous infection or seriously dysfunctional hepatic and renal reactions. The definition of persistent AF is when it lasts for more than 7 days and requires drugs or electric shocks to restore sinus rhythm. The diagnosis of congestive heart failure was based on the European Society of Cardiology criteria, including symptoms and/or signs of heart failure combined with abnormal systolic and diastolic function on echocardiography. Subsequently, AF-HF patients further were assigned into NYHA III and IV groups averagely on the basis of the functional classification system put forward by the New York Heart Association (NYHA). Patients with AF-HF included 7 (35%) reduced ejection fraction, 7 (35%) mid-ranged ejection fraction and 6 (30%) preserved heart failure. After fasting for 12 h, the whole blood was collected from each participant and saved in blood tubes vacuumly. The collected blood sample was immediately centrifuged at 1323×g for 10 min, the supernatant was for preservation and kept at − 80 °C condition before use.

### Proteomic LC-MS/MS analysis

Data independent acquisition (DIA) - based proteomics were performed by LC-MS/MS analysis. The study was carried out following such procedures: proteins extraction, quantification, detection, enzyme digestion and desalination, fraction separation and mass spectrometry, etc. Detailed experimental protocol of proteomics was provided in the Supplementary material.

### Proteomic data analysis

Fold change (FC) was calculated on the ratio of the mean quantization of HF group to AF group. Two-sided unpaired Welch’s t test was to judge the significance of each protein. Differentially expressed proteins (DEPs) were defined with the criteria of *p* value < 0.05 and FC > 1.5 or FC < 0.67. Mapping of DEPs and pathway analysis were achieved by KEGG pathway database [20–22]. The protein–protein interaction (PPI) network analysis of the DEPs was constructed from the STRING (https://string-db.org) and visualized by Cytoscape (version 3.7.1).

### Metabolomic LC-MS/MS analysis

Untargeted metabolomics were performed by LC-MS/MS analysis. The experimental procedures in the current study included metabolites extraction, LC-MS/MS detection, Data processing and metabolites identification, etc. Detailed experimental protocol of metabolomics was provided in the Supplementary material.

### Metabolomic data analysis

Partial least squares discriminant analysis (PLS-DA) was applied for the discrimination among AF patients with / without HF on metabolites effects. Variable importance in the projection (VIP) for each metabolite was deduced through the established model. The metabolites with VIP > 1 and *p* value < 0.05 and FC > 1.2 or FC < 0.83 were considered to be differentially expressed metabolites (DEMs). The metabolic pathways enrichment of DEMs was performed by KEGG database [20–22] .

### Correlation analysis of proteomics and metabolomics

The correlations between DEPs and DEMs were identified by Pearson correlation analysis. Based on *p* value < 0.05 and correlation coefficient > 0.5, a correlation network was constructed using Cytoscape (version 3.7.1). KEGG pathway enrichment analysis was conducted on DEPs and DEMs [20–22].

### Selection of biomarker panel by machine learning model

Random forest model was established to screen combined biomarkers of proteomics and metabolomics. The mean decrease accuracy and gini were calculated to evaluate the variable importance measures in the model. Based on 10-fold cross validation, random forest model and area under the receiver operating characteristic (AUC) analysis were used to evaluate predictive performance of biomarker panel.

### Statistical analysis

Continuous data were shown as means ± SD or median and interquartile range. Categorical data were shown as count and percentile. Student t test or Mann-Whitney test was used to compare the continuous variables between AF patients with and without HF. Chi-square test or Fisher’s exact test was used to analyze categorical data between two groups. *p* value < 0.05 were considered statistically significant. All the statistical analyses were performed using R (version 4.0.3).

## Results

### Overall design and clinical synopsis

**Fig. 1:**
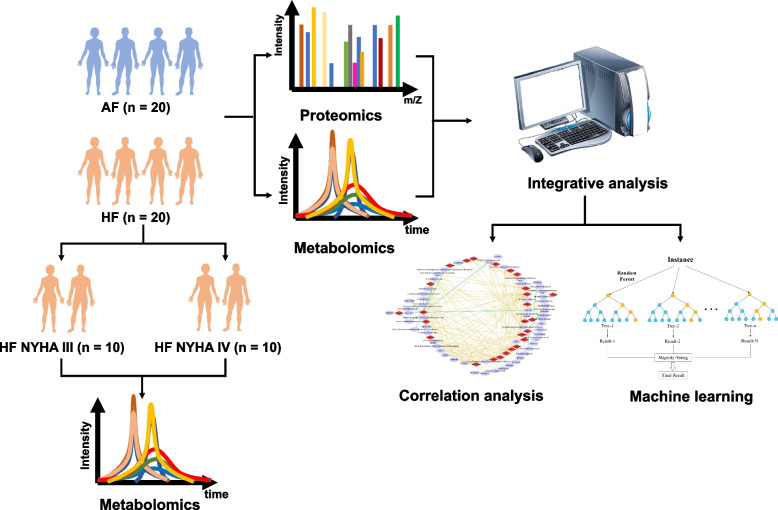
Flowchart of the study workflow

The study included 40 AF patients between the age of 45 and 85 years averaging 66.1. Among the 40 patients confirmed AF, 20 (50%) of them were presented with HF, half of which were with NYHA III stage and another half with NYHA IV stage (Fig. 1). Table S1 reveals specific demographic and clinical characteristics of the patients. The baseline characteristics were comparable among groups.

### Proteomic profiling of AF patients with / without HF

**Fig. 2:**
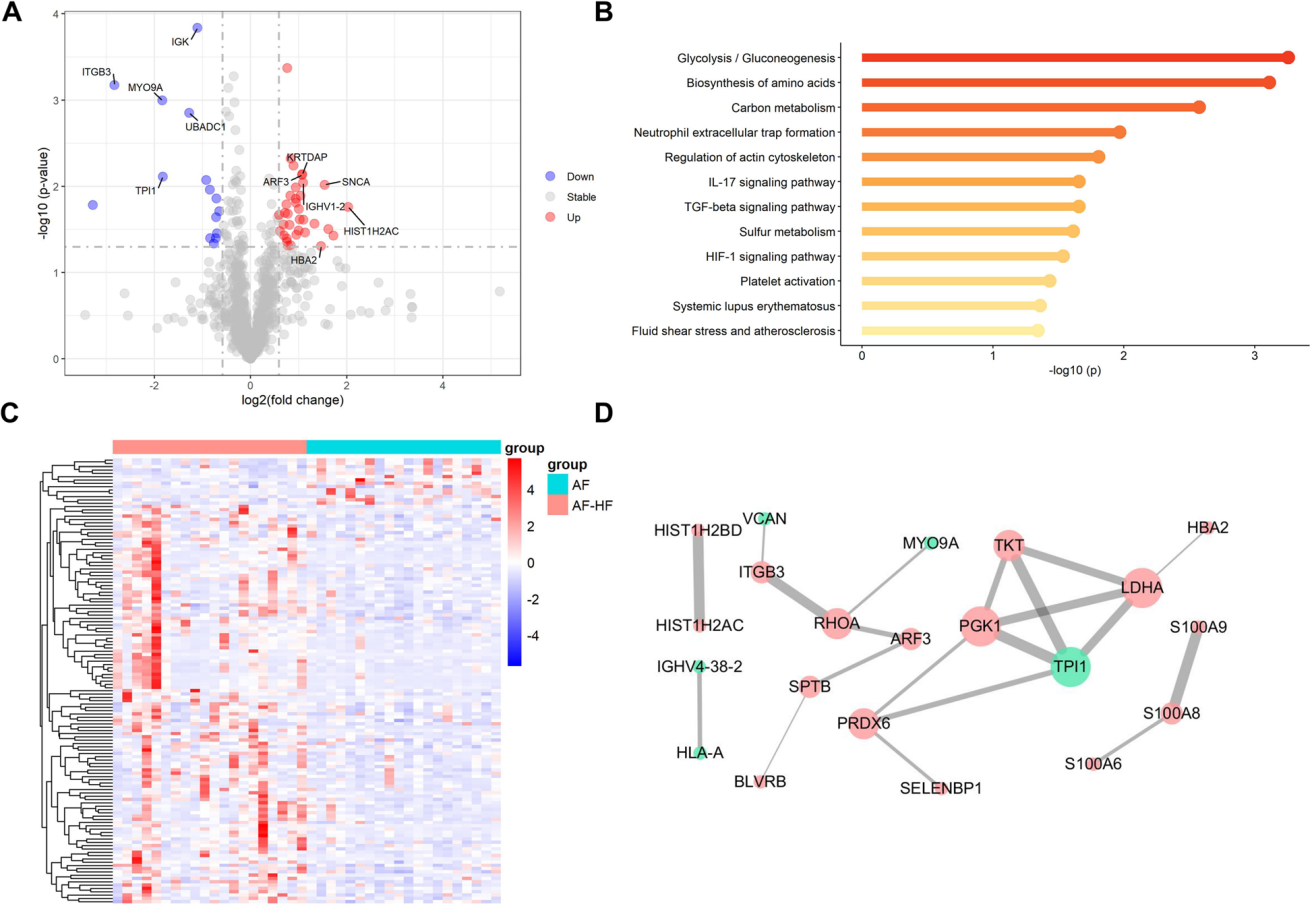
Proteomics analysis in patients with AF-HF compared to AF. The volcano plot (**A**) and heatmap (**B**) showed different proteins in AF compared AF-HF samples. **C** KEGG enrichment analysis of differently expressed proteins. **D** PPI network. Red represents the up-regulated proteins, as green for the opposite, respectively

A total of 50 proteins were identified with fold change (FC) > 1.5 or < 0.67 and *p* value < 0.05 (Fig. 2A) for discrimination AF patients with HF from those without HF. Of these DEPs, there were 35 up-regulated and 15 down-regulated proteins in HF patients compared to AF patients without HF (Fig. 2B). KEGG pathway enrichment analysis of significant DEPs revealed 12 significantly expressed metabolic pathways, such as Glycolysis/Gluconeogenesis, biosynthesis of amino acids, neutrophil extracellular trap formation, fluid shear stress and atherosclerosis, and platelet activation (Fig. 2C). The significantly enriched pathway of Glycolysis/Gluconeogenesis involved in TPI1, LDHA and PGK1. PPI analysis was performed with the 50 DEPs in the STRING database. A network containing 17 up-regulated proteins and 5 down-regulated proteins was constructed after removing unconnected nodes (Fig. 2D).

### Metabolomic profiling of AF patients with / without HF

**Fig. 3:**
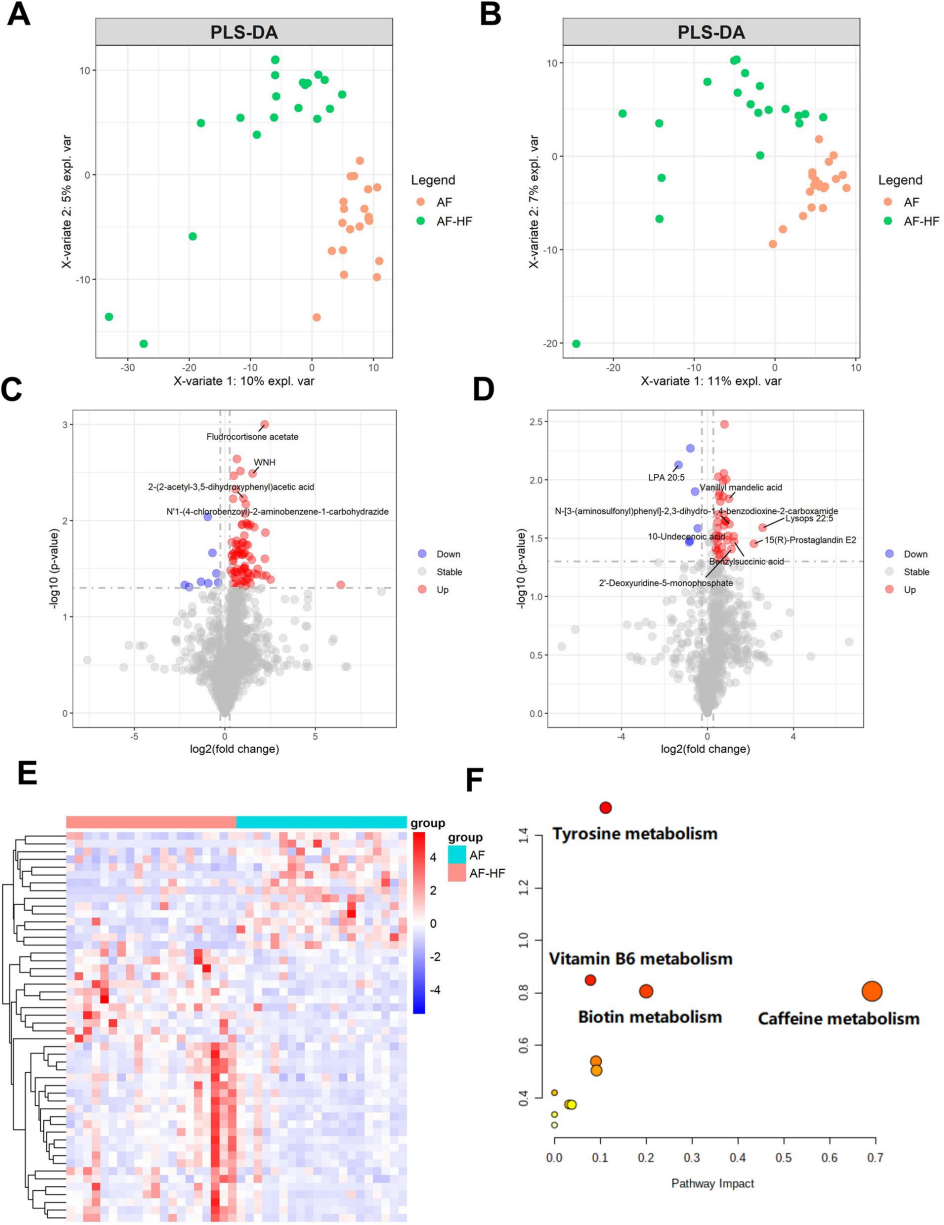
Metabolomic analysis in patients with AF-HF compared to AF. To distinguish AF-HF patients from without HF, PLSDA score plot were exhibited in positive ion (**A**) and negative ion (**B**). The volcano plot of positive ion (**C**) and negative ion (**D**) showed the metabolites between AF and AF-HF samples. (**E**) The heatmap showed the differently expressed metabolites in different samples. (**F**) Pathway analysis of differently expressed metabolites

The PLS-DA model was used to detect differences among AF patients with and without HF. The PLS-DA score plot revealed a clear separation between AF patients in the ESI (+ / -) mode (Fig. 3A, B). Under the condition of FC > 1.2 or FC < 0.83, *p* < 0.05 and VIP > 1, 90 and 45 metabolites were selected respectively by ESI (+ / -) mode as DEMs (Fig. 3C, D). Totally, 121 up-regulated and 14 down-regulated differential metabolites were discovered in HF patients compared to AF patients without HF (Fig. 3E). Then, the DEMs between AF patients with and without HF were used for metabolic pathway analysis. The mainly enriched pathway of Tyrosine metabolism involved in L-Dopa, Homovanillic acid (HVA) and Dopaquinone (Fig. 3F).

### Correlation analysis of proteomics and metabolomics in AF patients with / without HF

**Fig. 4:**
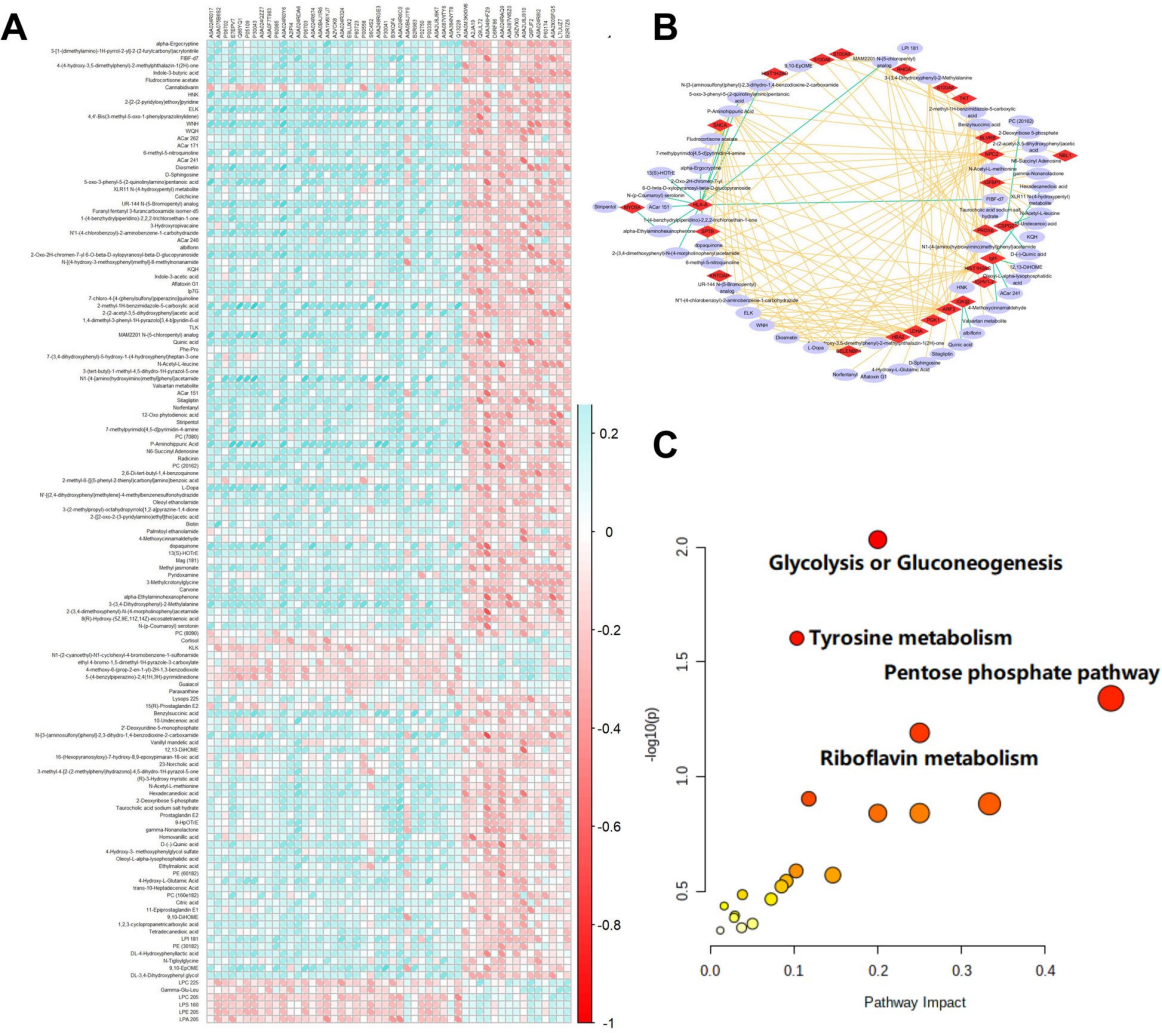
Integrated analysis of proteomics and metabolomics. **A** The correlation analysis of the known distinctionally expressed proteins and metabolites. **B** Proteins-metabolites correlation networks of differentially expressed proteins and metabolites. **C** Pathway analysis of differently expressed proteins and metabolites

We integrated datasets of DEPs and DEMs to explore a global correlation of proteomics and metabolomics in AF patients with / without HF. As a result, there was a strongly correlative patterns between the expressions of DEPs and DEMs (Fig. 4A). A correlation network was constructed between DEPs and DEMs with the cut-off value of correlation coefficient being 0.5. As a whole, the correlation network was mainly consisted of 30 DEPs and 61 DEMs in AF patients with / without HF (Fig. 4B). An integrated metabolic pathway analysis of proteomics and metabolomics was performed by MetaboAnalyst. Three pathways were significantly enriched at the significance level of 0.05, including Glycolysis or Gluconeogenesis; Tyrosine metabolism and Pentose phosphate pathway (Fig. 4C).

### Selection of biomarker panel using machine learning

**Fig. 5:**
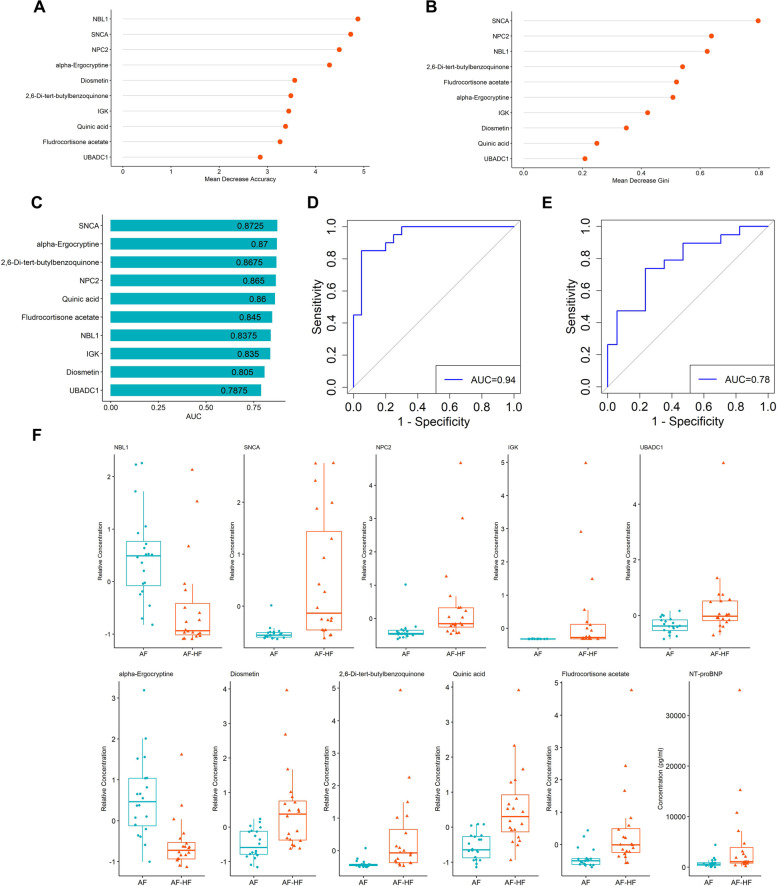
Biomarkers screening based on machine learning classifier. **A** Meandecrease accuracy of 10 candidate biomarkers in random forest analysis. **B** Mean decrease gini of 10 candidate biomarkers in random forest analysis. **C** Predictive performances of the 10 biomarkers. **D** ROC curve for prediction with the combined biomarkers. **E** ROC curve for prediction with NT-proBNP. **F** Relative concentrations of 10 biomarkers selected by random forest and concentration of NT-proBNP

To predict whether AF patients were with HF using proteomic and metabolomic data, we built a random forest machine learning model by the prioritization of 10 important variables. As presented, 5 proteins and 5 metabolites were selected as potential biomarkers (Fig. 5A, B). AUC analysis was to predict the performance of the biomarkers. The AUC values were as follows: UBADC1 (AUC = 0.7875), Quinic acid (AUC = 0.86), Diosmetin (AUC 0.805), IGK (AUC = 0.835), alpha-Ergocryptine (AUC = 0.87), Fludrocortisone acetate (AUC = 0.845), 2,6-Di-tert-butylbenzoquinone (AUC = 0.8675), NBL1 (AUC = 0.8375), NPC2 (AUC = 0.865), SNCA (AUC = 0.8725) (Fig. 5C). As expected, the combination of these 10 biomarkers implied high possibility of HF presence at the AUC of 0.94 (Fig. 5D). Notably, The AUC value of NT-proBNP was 0.78 for prediction of AF-HF (Fig. 5E). The relative concentrations of these 10 biomarkers and concentration of NT-proBNP between the two groups were shown in Fig. 5F.

### Subgroup analysis of HF classification

**Fig. 6:**
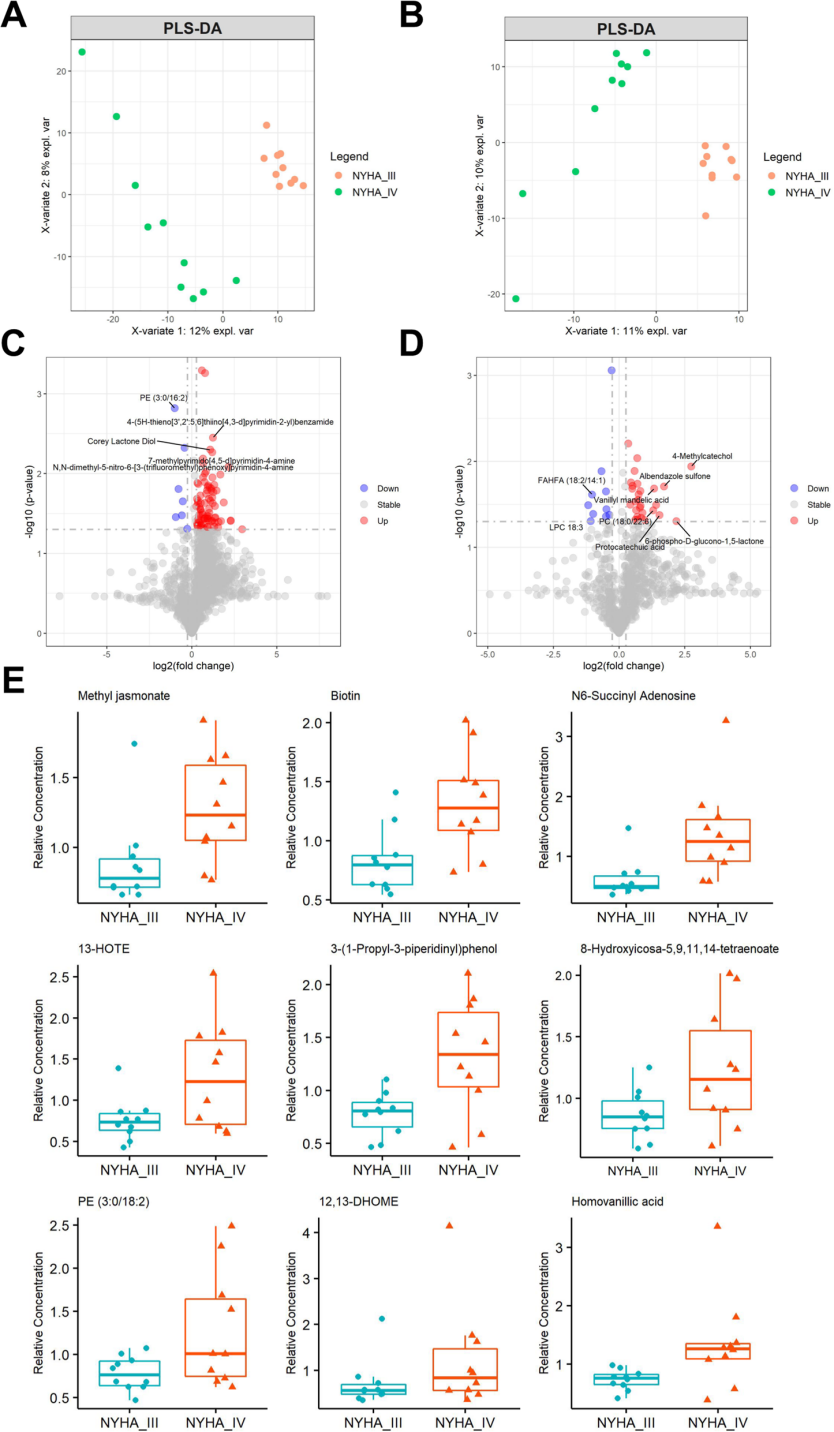
Metabolomic profiling analysis in AF-HF patients with NYHA IV compared to NYHA III. PLSDA score plot for discriminating HF patients with NYHA III and NYHA IV in positive ion (**A**) and negative ion (**B**). The volcano plot of positive ion (**C**) and negative ion (**D**) showed the metabolites between HF patients with NYHA III and NYHA IV. **E** Metabolite profiles of distinguishing NYHA III and NYHA IV

According to NYHA classification, HF patients were fitted into NYHA class III and NYHA class IV for subgroup analysis of metabolomics. The PLS-DA score plot revealed a clear separation between HF patients with class III and class IV in both the ESI+ mode and ESI- mode (Fig. 6A, B), 94 and 42 metabolites respectively, to discriminate NYHA class III from HF class IV. (Fig. 6C, D). We investigated 9 DEMs that can distinguish between AF and HF for HF classification, which involved in Methyl jasmonate, Biotin, N6-Succinyl Adenosine, 13-HOTE, 3-(1-Propyl-3-piperidinyl) phenol, 8-Hydroxyicosa-5,9,11,14-tetraenoate, PE (3:0/18:2), Homovanillic acid and 12,13-DHOME (Fig. 6E).

## Discussion

Arrhythmia and HF are both epidemics that commonly coexist and exacerbate each another, and AF is the most important factor of arrhythmia induced cardiomyopathies (AIC) in adults [23, 24]. The previous study had well elaborated the relations between AF and AIC. The related mechanisms of AF induced cardiomyopathies include: irregular rhythm, atrial systolic disfunction, and genetic factors. However, the specific molecular mechanism of AF induced HF is unclear. Recently, proteomics and metabolomics are a newly developing and novel tool not only to test for new risk factors but also to reveal underlying mechanisms in cardiovascular disease [18, 19]. In our study, we analyzed through the dysregulated molecules in AF with and without HF plasma samples and found remarkable changes in proteins and metabolites. The comprehensive multi-omics data indicated that characteristic pathways and dysregulated molecules in AF and AF-HF proteomic profiling or metabolomic profiling may help to reveal the potential biomarkers and underlying mechanism of AF induced HF.

Recent advances in high-throughput technologies make large-scale molecular profiling possible. Several studies had found some special genetics pattern in AF. However, relative to genetics, there are strong link between the proteomics or metabolomics and the phenotype of diseases. The proteomics or metabolomics can better display the occurrence and development of diseases. Several studies had found some metabolomics pattern in AF [25]. It was demonstrated that the mean ketone substrate during body metabolism such as beta-hydroxybutyrate increases along with ketogenic amino acids and glycine in AF [26]. Changes were also noticed in purine metabolic pathway by means of pathway enrichment analysis, as well as fatty acid metabolism in AF [27]. The pathological process of HF is accompanied by metabolic remodeling. The previous study identified novel risk biomarkers indicating left ventricular function weakened through metabolomics, indicating that under the impairment of LV-EF, patients show improved polyamine metabolite acisoga [28]. Furthermore, metabolic remodeling takes a vital role in cardiac remodeling. PKA and AMPK signaling crosstalk regulates metabolic remodeling in HF [29].

However, no elaborate understanding has been acquired about the patterns of the special proteomic and metabolomic in AF and AF-HF. In our study, we firstly analyzed AF patients to seek difference of proteomics in AF with HF. The results revealed 35 up-regulated and 15 down-regulated proteins in AF patients with HF compared to AF patients without HF. KEGG pathway enrichment analysis of significant DEPs indicated several noticeable metabolic pathways. Remarkably, Glycolysis/Gluconeogenesis pathway including TPI1, LDHA and PGK1 is the most significantly enriched pathway linked to HF. PPI analysis also showed a close interaction between the three proteins. Interestingly, the previous study reported a significantly change of Glycolysis/Gluconeogenesis pathway in HF by label-free shotgun proteomics approach [30]. It was suggested that the disordered Glycolysis/Gluconeogenesis pathway may be the pathogenic hub pathway for patients with AF to develop HF. Branched-chain amino acid metabolism are known for relating to the worsening adverse remodeling in the failing heart [31]. In our study, biosynthesis of amino acids was shown significantly altered in AF patients with HF, suggesting the synergistic effect of amino acid synthesis and metabolism on HF. In addition, significantly enriched Neutrophil extracellular trap formation pathway might be contribute to the pathogenesis of HF with preserved ejection fraction [32].

Meantime, we also analyzed AF and AF-HF plasma samples to evaluate the potential actions of metabolomics in AF-HF. We found that 121 up-regulated and 14 down-regulated differential metabolites were discovered in HF patients to those without HF. Then, the DEMs between AF patients with and without HF were used for metabolic pathway analysis. The mainly enriched pathway of Tyrosine metabolism involved in elevated levels of L-Dopa, HVA and Dopaquinone in AF patients with HF. In particular, HVA and Dopaquinone can be biosynthesized from L-Dopa, which was the precursor of dopamine [33]. As an endogenous catecholamine, dopamine worked as an autocrine and paracrine factor in the nonneuronal systems [34]. It has been reported that L-Dopa promotes dopamine release through D1 receptors [35], which has been proven to trigger ventricular arrhythmia in chronic HF [36]. Thus, the accumulation of L-Dopa, HVA and Dopaquinone in AF patients with HF might be associated with the facilitated regulation of dopamine and D1 receptors on HF.

We next integrated the proteomics and metabolomics datasets to generate a global view of plasma profiles. Several hub proteins and metabolites were presented with strong correlations. Three pathways were significantly enriched in integrated multi-omics, including Glycolysis/Gluconeogenesis, Tyrosine metabolism and Pentose phosphate pathway. Deoxyribose 5-phosphate and TKT, which are involved in Pentose phosphate pathway, were significantly up-regulated in AF with HF compared to AF without HF. Interestingly, certain anabolic pathways as amino acids and pentose phosphate were abundant when testing knockout mice of cardiomyocyte-restricted [36]. It has been proposed that the activation of Pentose phosphate pathway plays a critical role in regulating cellular oxidative stress [37]. Vimercati et.al indicated an important contribution of the oxidative pentose phosphate pathway activity to cardiac oxidative stress in HF [38]. Badolia et.al reported that during recovery, hearts could lead glycolytic metabolites into pentose-phosphate pathway, which can improve and protect the function of the heart by reducing oxidative stress [39]. In our study, we selected a panel of molecular signatures of proteins and metabolites associated with AF related HF using a machine learning model. A total of 5 proteins and 5 metabolites were screened as potential biomarkers, including UBADC1, IGK, NBL1, NPC2, SNCA, Quinic acid, Diosmetin, alpha-Ergocryptine, Fludrocortisone acetate and 2,6-Di-tert-butylbenzoquinone. Notably, these ten biomarkers altogether contributed an AUC value of 0.94, which could possibly predict for HF in AF.

NYHA functional class plays a central role in HF assessment, which has important implications for making individual clinical decisions [40]. Previous studies have reported amino acids and gut microbiota-dependent metabolites could discriminate early-stage HF from advanced-stage HF [41, 42]. However, few studies have systematically digged into the metabolic properties of AF related HF with NYHA class III and IV. In our study, we found nine of the identified DEMs that can distinguish between AF patients with and without HF also have the ability to distinguish NYHA class III from IV for HF in AF. In our study, HF patients were then divided into the two mentioned classes for subgroup analysis of metabolomics. A series of fatty acid metabolites, such as 12,13-diHOME, 13-HOTE, Methyl jasmonate and 8-Hydroxyicosa-5,9,11,14-tetraenoate were found to be up-regulated in AF-HF patients with NYHA class IV. Recent study has indicated that 12,13-diHOME promotes the uptake of altered lipid, as metabolism and presentation, and caused IL-10 secretion decreasing by human dendritic cells [43], suggested that the elevated 12,13-diHOME might be associated with altering immune cell metabolism. Pinckard et.al proposed that 12,13-diHOME was decreased in human patients with heart disease in a small population [44], but the study did not clarify whether the selected population was from HF or AF patients. Therefore, the exact molecular mechanism of 12,13-diHOME in AF patients with HF remains obscure and requires further certification. Overall, the 9 DEMs for discriminating the classification of HF deserve our attention, as well as later affirmance.

As we can find, a comprehensive insight of proteomic and metabolomic profiles of AF patients with and without HF was shown in this study. However, there may still be some limitations in our study. To start with, the restricted patient numbers may cause the precluding of parameters in certain proteins and metabolites as well as the sufficient statistical power needed to be improved. In addition, absolute quantitative proteomics and metabolomics techniques were not used in this study, so the molecules identified in this study need quantitative verification before being widely popularized to clinical application. Still, the majority of AF-HF patients in our study mainly included NYHA classification III and IV, so the multi-omic characteristics of AF-HF patients with early-stage have not been explored. Finally, this study mainly focused on the molecular profiles of HF induced by AF, so patients with sinus rhythm were not included. Thus, future studies of larger independent cohorts with multiple controls and quantitative multi-omic signatures with different HF-types and cardiomyopathy-types samples are needed to validate and complement the current findings.

## Conclusion

Through proteomic and metabolomic analyses, we picked out different proteins and metabolites in AF compared to AF-HF samples, and found that compared to AF-HF, those without HF differed in metabolic profiling by a large margin. So different types of patients could be distinguished using these two techniques as efficient molecular markers. In summary, novel apprehension to understand the mechanisms of AF-induced HF progression has been found through this study, as well as combining proteomics and metabolomics for the identification of novel factors for prognosis or treatment of AF with HF.

## Supplementary Information

**Additional file 1.**

## Data Availability

The datasets generated and analysed during the current study are available in this published article and the Supplementary information files.

## References

[1] Savarese G, Lund LH (2017). Global public health burden of heart failure. Card Fail Rev.

[2] Steffel J, Verhamme P, Potpara TS, Albaladejo P, Antz M, Desteghe L, Georg Haeusler K, Oldgren J, Reinecke H, Roldan-Schilling V (2018). The 2018 European heart rhythm association practical guide on the use of non-vitamin K antagonist oral anticoagulants in patients with atrial fibrillation: executive summary. Europace.

[3] Piccini JP, Hammill BG, Sinner MF, Hernandez AF, Walkey AJ, Benjamin EJ, Curtis LH, Heckbert SR (2014). Clinical course of atrial fibrillation in older adults: the importance of cardiovascular events beyond stroke. Eur Heart J.

[4] Miyasaka Y, Barnes ME, Gersh BJ, Cha SS, Bailey KR, Abhayaratna W, Seward JB, Iwasaka T, Tsang TS (2006). Incidence and mortality risk of congestive heart failure in atrial fibrillation patients: a community-based study over two decades. Eur Heart J.

[5] Sartipy U, Dahlstrom U, Fu M, Lund LH (2017). Atrial fibrillation in heart failure with preserved, mid-range, and reduced ejection fraction. JACC Heart Fail.

[6] McManus DD, Hsu G, Sung SH, Saczynski JS, Smith DH, Magid DJ, Gurwitz JH, Goldberg RJ, Go AS (2013). Cardiovascular research network PS: atrial fibrillation and outcomes in heart failure with preserved versus reduced left ventricular ejection fraction. J Am Heart Assoc.

[7] Wang TJ, Larson MG, Levy D, Vasan RS, Leip EP, Wolf PA, D'Agostino RB, Murabito JM, Kannel WB, Benjamin EJ (2003). Temporal relations of atrial fibrillation and congestive heart failure and their joint influence on mortality: the Framingham heart study. Circulation.

[8] Kotecha D, Piccini JP (2015). Atrial fibrillation in heart failure: what should we do?. Eur Heart J.

[9] Schnabel RB, Rienstra M, Sullivan LM, Sun JX, Moser CB, Levy D, Pencina MJ, Fontes JD, Magnani JW, McManus DD (2013). Risk assessment for incident heart failure in individuals with atrial fibrillation. Eur J Heart Fail.

[10] Carlisle MA, Fudim M, DeVore AD, Piccini JP (2019). Heart failure and atrial fibrillation, like fire and fury. JACC Heart Fail.

[11] Pandey A, Mann M (2000). Proteomics to study genes and genomes. Nature.

[12] Nicholson JK, Lindon JC (2008). Systems biology: Metabonomics. Nature.

[13] Gstaiger M, Aebersold R (2009). Applying mass spectrometry-based proteomics to genetics, genomics and network biology. Nat Rev Genet.

[14] Ong SE, Mann M (2005). Mass spectrometry-based proteomics turns quantitative. Nat Chem Biol.

[15] Tyanova S, Temu T, Cox J (2016). The MaxQuant computational platform for mass spectrometry-based shotgun proteomics. Nat Protoc.

[16] Chen ZZ, Gerszten RE (2020). Metabolomics and proteomics in type 2 diabetes. Circ Res.

[17] Nayor M, Brown KJ, Vasan RS (2021). The molecular basis of predicting atherosclerotic cardiovascular disease risk. Circ Res.

[18] Hu B, Ge W, Wang Y, Zhang X, Li T, Cui H, Qian Y, Zhang Y, Li Z (2021). Metabolomic and proteomic analyses of persistent Valvular atrial fibrillation and non-Valvular atrial fibrillation. Front Genet.

[19] Li MY, Chen HX, Hou HT, Wang J, Liu XC, Yang Q, He GW (2021). Biomarkers and key pathways in atrial fibrillation associated with mitral valve disease identified by multi-omics study. Ann Transl Med.

[20] Kanehisa M, Goto S (2000). KEGG: Kyoto encyclopedia of genes and genomes. Nucleic Acids Res.

[21] Kanehisa M (2019). Toward understanding the origin and evolution of cellular organisms. Protein Sci.

[22] Kanehisa M, Furumichi M, Sato Y, Ishiguro-Watanabe M, Tanabe M (2021). KEGG: integrating viruses and cellular organisms. Nucleic Acids Res.

[23] Gopinathannair R, Etheridge SP, Marchlinski FE, Spinale FG, Lakkireddy D, Olshansky B (2015). Arrhythmia-induced cardiomyopathies: mechanisms, recognition, and management. J Am Coll Cardiol.

[24] Calo L, De Ruvo E, Sette A, Sciarra L, Scioli R, Sebastiani F, Topai M, Iulianella R, Navone G, Lioy E (2007). Tachycardia-induced cardiomyopathy: mechanisms of heart failure and clinical implications. J Cardiovasc Med (Hagerstown).

[25] Jung Y, Cho Y, Kim N, Oh IY, Kang SW, Choi EK, Hwang GS (2018). Lipidomic profiling reveals free fatty acid alterations in plasma from patients with atrial fibrillation. PLoS One.

[26] Mayr M, Yusuf S, Weir G, Chung YL, Mayr U, Yin X, Ladroue C, Madhu B, Roberts N, De Souza A (2008). Combined metabolomic and proteomic analysis of human atrial fibrillation. J Am Coll Cardiol.

[27] Zhou J, Sun L, Chen L, Liu S, Zhong L, Cui M (2019). Comprehensive metabolomic and proteomic analyses reveal candidate biomarkers and related metabolic networks in atrial fibrillation. Metabolomics.

[28] Puetz A, Artati A, Adamski J, Schuett K, Romeo F, Stoehr R, Marx N, Federici M, Lehrke M, Kappel BA (2022). Non-targeted metabolomics identify polyamine metabolite acisoga as novel biomarker for reduced left ventricular function. ESC Heart Fail.

[29] Chaanine AH, Higgins L, Markowski T, Harman J, Kachman M, Burant C, Navar LG, Busija D, Delafontaine P (2021). Multi-omics approach profiling metabolic remodeling in early systolic dysfunction and in overt systolic heart failure. Int J Mol Sci.

[30] Dai DF, Hsieh EJ, Chen T, Menendez LG, Basisty NB, Tsai L, Beyer RP, Crispin DA, Shulman NJ, Szeto HH (2013). Global proteomics and pathway analysis of pressure-overload-induced heart failure and its attenuation by mitochondrial-targeted peptides. Circ Heart Fail.

[31] Karwi QG, Lopaschuk GD. Branched-chain amino acid metabolism in the failing heart. Cardiovasc Drugs Ther. 2022:1–8. 10.1007/s10557-022-07320-4. 10.1007/s10557-022-07320-4 35150384

[32] Zhang XL, Wang TY, Chen Z, Wang HW, Yin Y, Wang L, Wang Y, Xu B, Xu W (2022). HMGB1-promoted neutrophil extracellular traps contribute to cardiac diastolic dysfunction in mice. J Am Heart Assoc.

[33] Orrillo SJ, de Dios N, Asad AS, De Fino F, Imsen M, Romero AC, Zarate S, Ferraris J, Pisera D (2020). Anterior pituitary gland synthesises dopamine from l-3,4-dihydroxyphenylalanine (l-dopa). J Neuroendocrinol.

[34] Yamaguchi T, Sumida TS, Nomura S, Satoh M, Higo T, Ito M, Ko T, Fujita K, Sweet ME, Sanbe A (2020). Cardiac dopamine D1 receptor triggers ventricular arrhythmia in chronic heart failure. Nat Commun.

[35] Viaro R, Longo F, Vincenzi F, Varani K, Morari M (2021). L-DOPA promotes striatal dopamine release through D1 receptors and reversal of dopamine transporter. Brain Res.

[36] Fernandez-Caggiano M, Kamynina A, Francois AA, Prysyazhna O, Eykyn TR, Krasemann S, Crespo-Leiro MG, Vieites MG, Bianchi K, Morales V (2020). Mitochondrial pyruvate carrier abundance mediates pathological cardiac hypertrophy. Nat Metab.

[37] Ren Y, Shen HM (2019). Critical role of AMPK in redox regulation under glucose starvation. Redox Biol.

[38] Vimercati C, Qanud K, Mitacchione G, Sosnowska D, Ungvari Z, Sarnari R, Mania D, Patel N, Hintze TH, Gupte SA (2014). Beneficial effects of acute inhibition of the oxidative pentose phosphate pathway in the failing heart. Am J Physiol Heart Circ Physiol.

[39] Badolia R, Ramadurai DKA, Abel ED, Ferrin P, Taleb I, Shankar TS, Krokidi AT, Navankasattusas S, McKellar SH, Yin M (2020). The role of nonglycolytic glucose metabolism in myocardial recovery upon mechanical unloading and circulatory support in chronic heart failure. Circulation.

[40] Miller RJ, Howlett JG, Exner DV, Campbell PM, Grant AD, Wilton SB (2015). Baseline functional class and therapeutic efficacy of common heart failure interventions: a systematic review and Meta-analysis. Can J Cardiol.

[41] Aquilani R, La Rovere MT, Corbellini D, Pasini E, Verri M, Barbieri A, Condino AM, Boschi F (2017). Plasma amino acid abnormalities in chronic heart failure. Mechanisms, potential risks and targets in human myocardium metabolism. Nutrients.

[42] Troseid M, Ueland T, Hov JR, Svardal A, Gregersen I, Dahl CP, Aakhus S, Gude E, Bjorndal B, Halvorsen B (2015). Microbiota-dependent metabolite trimethylamine-N-oxide is associated with disease severity and survival of patients with chronic heart failure. J Intern Med.

[43] Levan SR, Stamnes KA, Lin DL, Panzer AR, Fukui E, McCauley K, Fujimura KE, McKean M, Ownby DR, Zoratti EM (2019). Elevated faecal 12,13-diHOME concentration in neonates at high risk for asthma is produced by gut bacteria and impedes immune tolerance. Nat Microbiol.

[44] Pinckard KM, Shettigar VK, Wright KR, Abay E, Baer LA, Vidal P, Dewal RS, Das D, Duarte-Sanmiguel S, Hernandez-Saavedra D (2021). A novel endocrine role for the BAT-released Lipokine 12,13-diHOME to mediate cardiac function. Circulation.

