# Supplementary material for: Integration of proteomic and metabolomic characterization in atrial fibrillation-induced heart failure
Source: BMC Genomics. 2022 Dec 1;23:789. doi: 10.1186/s12864-022-09044-z (PMC9714089; doi:10.1186/s12864-022-09044-z)
Supplement: Supplementary file 1 — Additional file 1. [file 12864_2022_9044_MOESM1_ESM.zip › Supplementary methods and materials-revised 10-27.docx]

**Supplementary Methods materials**

**Proteomic Analysis**

**Proteins Extraction and Quality Test**

Plasma sample and proteominer beads (BioRAD, USA) were added to a 1.5 ml micro tube, mixed for 2 h, and centrifuged at 10,000 g for 5 min at 4 ℃. The beads were washed with wash buffer and 0.4 ml 1 % TFA for 10 min. The supernatant was then freezed into a dry powder, and was subsequently dissolved with dissolution buffer (8 M Urea, 100 mM TEAB, pH 8.5). The buffer was diminished with 10 mM DTT for 1 h at 56 ℃, and subsequently alkylated for 1 h. BSA standard protein solutions (20 µL, 0 to 0.5 g/L) and sample (20 µL) were added into 96-well plate. G250 dye solution (180 µL) was added into plate for 5 min, the absorbance was detected at 595 nm. For analysis, 20 µg of the sample was separated by electrophoresis, using 12% SDS-PAGE gel.

**Trypsin Treatment**

Sample was filled with DB lysis buffer as mentioned above, to 100 µL. Then the sample was digested by trypsin and 100 mM TEAB buffer at 37 °C for 4 h. After adding CaCl_2_, sample was digested overnight. To maintain pH Above 3, formic acid was then added before centrifuging at 12,000 g for 5 min. The supernatant was gained and poured to C18 column. The eluents (using elution buffer: 0.1% formic acid, 70% acetonitrile) of each sample were directly lyophilized.

**Separation of Fractions**

Mobile phase A (2% acetonitrile, pH 10.0) and B (98% acetonitrile) were used to dispense a gradient elution. After dissolved in solution A, the lyophilized powder was then centrifuged. The sample was separated by HPLC system (Rigol L3000) using a C18 column, while the temperature of the column oven was 45 °C. The eluates were monitored at the UV wavelength of 214 nm, and finally combined into 4 fractions after collecting one tube per minute. All fractions were dried vacuumly, which then, were redissolved in 0.1% formic acid adjusted by water.

**LC-MS/MS Analysis**

Shotgun proteomics analyses were carried out under EASY-nLCTM 1200 UHPLC system (Thermo Fisher) combined with a Q ExactiveTM HF-X mass spectrometer (Thermo Fisher) in data-dependent acquisition (DDA) mode for the development of the transition library. Sample was injected into a home-made C18 Nano-Trap column (4.5 cm × 75 μm, 3 μm) containing 4 μg fraction supernatant and 0.8 μL iRT reagent. Peptides were linear gradient eluted by the home-made analytical column (15 cm × 150 μm, 1.9 μm). The samples were evaluated using a Thermo Fisher Q ExactiveTM HF-X mass spectrometer with a Nanospray FlexTM (ESI) ion source, a spray voltage of 2.1 kV, and a 320 °C ion transport capillary temperature. The full scan extended from 350 to 1500 m/z, with a resolution of 120000 (at m/z 200), an automated gain control (AGC) target value of 3106, and an ion injection duration of 80 ms. The top 40 most abundant precursors were chosen and fragmented by higher energy collisional dissociation (HCD) before being examined by MS/MS. The resolution was 15000 (at m/z 200), the automatic gain control (AGC) target value was 5×10^4^ , and the maximum ion injection time was 45 ms. A normalized collision energy of 27%, an intensity threshold of 1.1104, and a dynamic exclusion parameter of 20 seconds are also included. The raw MS detection data was designated ".raw" and was used to create the DDA spectrum library.

Mobile phase A (0.1% FA dissolved in H_2_O) and B (same content in 80% ACN) were used to dispense a gradient elution. Sample was injected into the EASY-nLCTM 1200 UHPLC system, which included 4 g fraction supernatant and 0.8 l iRT reagent (Thermo Fisher). The system was coupled with an Orbitrap Q ExactiveTM HF-X mass spectrometer (Thermo Fisher), which was operated in the data-independent acquisition (DIA) mode with the spray voltage of 2.1 kV, Nanospray Flex™（ESI） and capillary temperature of 320 °C. The m/z ranged from 350 to 1500 for the acquisition of DIA. MS1 resolution was set to 60000 (at m/z 200), full scan AGC target value was 5×10^5.^ Peptides were fragmented by HCD in MS2, the resolution of which was set to 30000 (at 200 m/z), and AGC target value was 1×10^6^, a normalized collision energy of 27%.

**Identification and Quantitation of Protein**

The engine (Proteome Discoverer 2.2, Thermo) searched the resulting spectra of each fraction separately against Human Uniport database. Mass tolerance for precursor ion was 10 ppm and 0.02 Da for product ion were set as search parameters. Carbamidomethyl, oxidation of methionine (M) and acetylation were separately set as fixed dynamic and N-Terminal modifications in PD 2.2. There could be at most two missed distinctive sites. In order to analyze more efficiently, more parameters were set during retrieval: Peptide Spectrum Matches (PSMs) were identified with the credibility of over 99%, and identified proteins were also remained when FDR < 1.0%, which included more than one original peptide. The identified protein contained at least 1 unique peptide. The result of search and identification by PD 2.2 software was implanted into Spectronaut (version 14.0, Biognosys) software to structure a library. After setting peptides and ion pair selection rules, the eligible peptides and product ions were selected out of spectrum, and then generate a Target List. To which the DIA data was imported, while the ion-pair chromatographic peaks were extracted. To assess the peptides qualitatively and quantitatively, the ion and calculating peak area was matched. The iRT was added to the sample to correct retention time, with a precursor ion q-value cutoff of 0.01. A total of 1526 quantified proteins were detected in one or more of the 40 plasma samples. Of these proteins 1026 had ≤50% missingness in all 40 samples and were finally included in the subsequent analysis. Missing values for the unambiguous proteins with ≤ 50% missingness were imputed using K-Nearest Neighbor method.

**Metabolomic Analysis**

**Metabolites Extraction**

After a resuspend with prechilled methanol (80%) and formic acid (0.1%) by well vortex, 100 μL of the samples precleared by centrifugation at 15,000 g, 4 °C for 20 min. After diluted to 53% methanol, some of the supernatant were centrifuged at 15,000 g, 4 °C for 20 min.

**LC-MS/MS Analysis**

The Vanquish UHPLC system (Thermo Fisher, Germany), coupled with an Orbitrap Q ExactiveTMHF-X mass spectrometer (Thermo Fisher, Germany) by Novogene Co., Ltd. (Beijing, China) carried out the LC-MS/MS analyses. Samples were injected onto a Hypesil Gold column (100 × 2.1 mm, 1.9 μm) using a 17-min linear gradient at a flow rate of 0.2 mL / min. Different eluents were perfused to polarities: eluent A (0.1% FA) and eluent B (methanol) of positive polarity, as eluent A (5 mM ammonium acetate, pH 9.0) and eluent B (Methanol) of negative polarity. The solvent gradient was established as follows: 2% B, 1.5 min; 2-100% B, 12.0 min; 100% B, 14.0 min; 100-2% B, 14.1 min; 2% B, 17 min. The spray voltage was 3.2 kV, the capillary temperature was 320 °C, the sheath gas flow rate was 40 arb, and the aux gas flow rate was 10 arb on the Q ExactiveTMHF-X mass spectrometer in both polarity mode.

**Data processing and metabolite identification**

Using Compound Discovery 3.0 (CD 3.0, Thermo Fisher), raw files generated by UHPLC-MS/MS were aligned, picked, and quantified for each metabolite using the raw data files. The main parameters were set as follows: retention time tolerance, 0.2 minutes; actual mass tolerance, 5ppm; signal intensity tolerance, 30%; signal/noise ratio, 3; and minimum intensity, et al. After that, peak intensities were normalized to the total spectral intensity. Based on normalized spectrum data, additive ion, fragment ion, and molecular ion peak formulas were predicted. In order to obtain accurate qualitative and quantitative results, peaks were matched to mzCloud, mzVault, and MassList databases. A total of 1505 ions in ESI+ mode and 798 ions in ESI− mode were identified for subsequent statistical analysis.

**Table S1 Detailed demographic and clinical characteristics of AF patients**

| Variables | AF (*n* = 20) | HF (*n* = 20) | *p*-value | HF NYHA class III (*n* = 10) | HF NYHA class IV (*n* = 10) | *p*-value |
| --- | --- | --- | --- | --- | --- | --- |
| Sex - no. (%) |  |  | 0.3272 |  |  | 1 |
| Female | 6 (30) | 9 (45) |  | 5 (50) | 4 (40) |  |
| Male | 14 (70) | 11 (55) |  | 5 (50) | 6 (60) |  |
| Age (year) | 65.25 ± 8.6 | 66.95 ± 11.86 | 0.6068 | 63.90 ± 12.49 | 70.00 ±10.97 | 0.261 |
| Smoke - no. (%) | 9 (45) | 8 (40) | 0.7491 | 5 (50) | 3 (30) | 0.6499 |
| Alcohol - no. (%) | 5 (25) | 5 (25) | 1 | 2 (20) | 3 (30) | 1 |
| Hypertension - no. (%) | 12 (60) | 11 (55) | 0.7491 | 4 (40) | 7 (70) | 0.3698 |
| Diabetes - no. (%) | 7 (35) | 11 (55) | 0.2036 | 6 (60) | 5 (50) | 1 |
| Hyperlipemia - no. (%) | 10 (50) | 5 (25) | 0.1025 | 2 (20) | 3 (30) | 1 |
| Ischemic cardiomyopathy - no. (%) | 9 (45) | 9 (45) | 1 | 4 (40) | 5 (50) | 1 |
| Dilated cardiomyopathy - no. (%) | 0 (0) | 3 (15) | 0.2308 | 2 (20) | 1 (10) | 1 |
| Valvular disease - no. (%) | 2 (10) | 4 (20) | 0.6614 | 3 (30) | 1 (10) | 0.5820 |
| Medication use |  |  |  |  |  |  |
| Anticoagulant - no. (%) | 18 (90) | 17 (85) | 1 | 8 (80) | 9 (90) | 1 |
| Antiarrhythmic - no. (%) | 5 (25) | 4 (20) | 1 | 2 (20) | 2 (20) | 1 |
| Loop diuretic - no. (%) | 0 (0) | 1 (5) | 1 | 1 (10) | 0 (0) | 1 |
| Digitalis - no. (%) | 1 (5) | 6 (30) | 0.0915 | 3 (30) | 3 (30) | 1 |
| Beta-blocker - no. (%) | 12 (60) | 13 (65) | 0.744 | 6 (60) | 7 (70) | 1 |
| Antiplatelet - no. (%) | 6 (30) | 3 (15) | 0.4506 | 0 (0) | 3 (30) | 0.2105 |
| Laboratory measuers |  |  |  |  |  |  |
| NT-proBNP (pg/ml) | 595.6 (325- 894.2) | 1076 (713.3- 4671) | 0.0046 | 999.75 (713.3-2048) | 2585 (1076-10749) | 0.1914 |
| Red blood cell (10^12^/L) | 4.78 ± 0.45 | 4.45 ± 0.65 | 0.0696 | 4.71 ± 0.7 | 4.19 ± 0.49 | 0.0708 |
| White blood cell (10^9^/L) | 7.09 ± 1.45 | 6.88 ± 1.92 | 0.6972 | 6.76 ± 1.41 | 7 ± 2.41 | 0.7879 |
| PLT (10^9^/L) | 220.91 ± 46.38 | 210.25 ± 58.43 | 0.527 | 198.62 ± 20.86 | 221.89 ± 80.45 | 0.3964 |
| HDL - C (mmol/L) | 1.22 ± 0.21 | 1.26 ± 0.34 | 0.6405 | 1.18 ± 0.28 | 1.35 ± 0.39 | 0.264 |
| LDL - C (mmol/L) | 2.74 ± 0.62 | 2.84 ± 0.67 | 0.6237 | 2.83 ± 0.59 | 2.86 ± 0.78 | 0.9382 |
| TC (mmol/L) | 4.69 ± 1.03 | 4.71 ± 0.9 | 0.9494 | 4.62 ± 0.77 | 4.8 ± 1.07 | 0.6872 |
| TG (mmol/L) | 2.26 ± 1.17 | 1.69 ± 1.21 | 0.1432 | 2.07 ± 1.55 | 1.26 ± 0.45 | 0.1509 |
| Glucose (mg/dL) | 6.13 ± 1.56 | 6.36 ± 1.43 | 0.6395 | 6.53 ± 1.48 | 6.16 ± 1.43 | 0.5826 |
| LVEF (median, %) | 61.55 ± 10.36 | 45.8 ± 15.42 | 0.0005 | 53.3 ± 11.52 | 38.3 ± 15.64 | 0.0251 |

PLT: Platelet Count, HDL-C: High Density Liptein Cholesterol, LDL-C: Low Density Lipoprotein Cholesterol, TC: Serum Total Cholesterol, TG: Triglyceride, LVEF: Left Ventricular Ejection Fractions

**Table S2 Identified metabolites showing statistically significant changes between AF-HF patients with NYHA class III and IV**

| Compound | Class | p-value ^a^ | FC ^b^ | VIP ^c^ | Retention time (min) | Measured mass (Da) | ESI mode |
| --- | --- | --- | --- | --- | --- | --- | --- |
| Methyl jasmonate | Fatty Acyls | 0.023315 | 1.439399 | 1.62115 | 11.839 | 225.1493 | negative |
| 13-HOTE | Fatty Acyls | 0.036553 | 1.69074 | 1.549772 | 13.982 | 295.2281 | positive |
| 8-Hydroxyicosa-5,9,11,14-tetraenoate | Fatty Acyls | 0.044424 | 1.436865 | 1.56199 | 14.612 | 321.2416 | positive |
| 12,13-DHOME | Fatty Acyls | 0.028115 | 1.673933 | 1.809596 | 12.588 | 313.2401 | positive |
| 3-(1-Propyl-3-piperidinyl)phenol | Piperidines | 0.012772 | 1.694107 | 1.822738 | 12.893 | 220.1706 | positive |
| N6-Succinyl Adenosine | Purine nucleosides | 0.015456 | 2.227639 | 1.757491 | 6.559 | 384.1158 | negative |
| Biotin | Biotin and derivatives | 0.007511 | 1.593955 | 1.877576 | 5.728 | 245.0967 | negative |
| Homovanillic acid | Phenols | 0.039779 | 1.853468 | 1.654514 | 3.315 | 181.0512 | positive |
| PE (3:0/18:2) | Phosphatidyl ethanolamines | 0.047431 | 1.636536 | 1.693998 | 14.588 | 532.3065 | positive |

^a^ *p*-values were derived from two-tailed Welch’s t-test.

^b^ Fold change was calculated from the arithmetic mean values of each group. Fold change with a positive value indicates that the concentration of certain metabolite is up-regulated in AF-HF patients with NYHA class IV compared to NYHA class III.

^c^ VIP was calculated based on the PLS-DA model.
